# Evaluating Anti-CCL25 as a Therapeutic Strategy to Disrupt Foci Formation in a Spontaneous Murine Model of Sjögren’s Disease

**DOI:** 10.3390/ijms26188802

**Published:** 2025-09-10

**Authors:** Martha Tsaliki, Biji T. Kurien, Joshua Cavett, John A. Ice, Kristi A. Koelsch, Robert Hal Scofield

**Affiliations:** 1 Arthritis & Clinical Immunology Research Program, Oklahoma Medical Research Foundation, 825 NE 13th Street, Oklahoma City, OK 73104, USA; 2 Department of Pathology, University of Oklahoma Health Sciences Center, 940 Stanton L. Young Blvd, Oklahoma City, OK 73104, USA; 3 Department of Veterans Affairs Medical Center, 921 NE 13th Street, Oklahoma City, OK 73104, USA; 4 Department of Medicine, College of Medicine, University of Oklahoma Health Sciences Center, 940 Stanton L. Young Blvd, Oklahoma City, OK 73104, USA

**Keywords:** Sjögren’s disease, CCR9+ T cells, CCL25, salivary gland inflammation, mouse model, autoimmunity

## Abstract

Sjögren’s disease (SjD) targets the salivary and lacrimal glands and is characterized by autoantibody production and glandular lymphocytic infiltrate with ectopic germinal centers (EGCs). The chemokine CCL25 recruits CCR9^+^ CD4^+^ T cells to the salivary glands to promote B cell activation. However, the therapeutic potential of targeting the CCL25–CCR9 axis to limit glandular inflammation and lymphoid neogenesis remains largely unexplored. Evaluate whether blocking the CCL25–CCR9^+^ T cell axis with a monoclonal antibody could reduce immune infiltration, ectopic germinal center (EGC) formation, and local autoantibody production in the NOD.H2(h4) mouse model of SjD. Female NOD.H2(h4) mice were administered anti-CCL25 antibody, isotype control, or PBS intraperitoneally for 12 weeks. Sera and saliva were collected to evaluate anti-Ro52 antibodies via ELISA across treatment groups. Salivary glands were harvested and processed for H&E staining to assess lymphocytic infiltration and focus scores. Treatment with α-CCL25 was well tolerated, with no significant differences in body weight or stimulated salivary flow between treatment groups. Histopathological evaluation revealed no reduction in lymphocytic infiltration, focus scores, or percentage of inflamed tissue in α-CCL25-treated mice compared to controls. Anti-Ro52 antibodies were undetectable in plasma or saliva across all groups and timepoints. Systemic CCL25 blockade did not significantly alter salivary gland inflammation, function, or autoantibody production in NOD.H2(h4) mice. These findings suggest that monotherapy targeting the CCL25–CCR9 axis may be insufficient to resolve glandular autoimmunity in this model and that additional or combinatorial strategies may be necessary for effective intervention.

## 1. Introduction

Sjögren’s disease (SjD) is a chronic autoimmune exocrinopathy disease that is primarily characterized by lymphocytic involvement of the lacrimal and salivary glands and extensive dryness of the mouth and eyes [1] Some SjD patients experience systemic disease, developing debilitating extraglandular complications involving many organ systems that significantly impact their quality of life [1]. Sjögren’s disease is among the most common rheumatic diseases after rheumatoid arthritis and affects approximately 1% of the world population, with postmenopausal women comprising the highest-risk group [1,2]. Patients are typically treated with the goal of alleviating symptoms through the application of artificial saliva and/or tears, anti-inflammatory eye drops, pain relievers, cholinergic agents, glucocorticoids, and antifungal agents [3].

One histopathological feature of SjD salivary glands is the presence of ectopic germinal center-like structures (EGCs), which are most commonly observed in patients with severe disease [4]. These lymphoid structures mirror the architecture of secondary lymphoid organ germinal centers and are found in approximately 25% of labial salivary gland biopsies from SjD patients [4]. The presence of EGCs is associated with higher lymphocytic focus scores, elevated serum levels of anti-Ro (or SSA) and anti-La (or SSB) autoantibodies, increased expression of cytokines such as IL-21 and IFN-γ, and an increased risk of non-Hodgkin lymphomas [4,5]. Functionally, EGCs create a local microenvironment that supports antigen-driven B cell selection, somatic hypermutation, class switching, and the differentiation of B cells into antibody-secreting plasma cells or memory B cells [5,6]. The formation of EGCs within glandular tissue indicates that local autoreactive immune responses may be both initiated and maintained directly at the site of exocrine gland damage [4,5].

The immunopathology of salivary glands in SjD is multifactorial, involving contributions from both the epithelium and various immune cell types [7,8,9]. Together, these factors drive immune cell infiltration and promote the formation and organization of EGCs [7,8,9]. Among the key immune cell populations involved are follicular helper T (Tfh) cells, which orchestrate local B cell activation within the salivary glands [10,11]. Canonical Tfh cells are defined by the expression of CXCR5, PD-1, and ICOS, and in SjD patients these cells are typically increased in both peripheral blood and glandular tissues [11]. In SjD, Tfh cells are closely associated with autoantibody production (anti-Ro), lymphocytic focus scores, and elevated expression of IL-21, a cytokine critical for germinal center function and plasma cell differentiation [10,11]. In parallel, peripheral helper T (Tph) cells, which lack CXCR5 but express high levels of PD-1, ICOS, and IL-21, are also significantly enriched in both the peripheral circulation and salivary glands of SjD patients [10]. These Tph cell counts correlate with clinical disease activity, as measured by the European Alliance of Associations for Rheumatology (EULAR) Sjögren’s Syndrome Disease Activity Index (ESSDAI) [10]. Tph cells are also linked to higher numbers of antibody-secreting B cells in the salivary glands, supporting local B cell maturation and antibody production outside of traditional follicular pathways and contributing to ectopic germinal center-like activity [10].

Another CD4^+^ T cell subset that plays a crucial role in SjD pathogenesis is the CCR9^+^ population [7]. These cells migrate into inflamed salivary gland tissues in response to CCL25, a chemokine that is overexpressed by glandular epithelial cells in SjD and selectively recruits CCR9^+^ CD4^+^ T cells [7,8]. While Tph and CCR9^+^ Tfh-like cells share some functional features, they represent non-overlapping subsets [11]. CCR9^+^ Tfh-like cells (CXCR5^−^CCR9^+^) produce IL-21 and IFN-γ, express PD-1 and ICOS, and are significantly increased in the periphery of SjD patients, particularly those with anti-Ro antibodies [11]. Notably, the CXCR5^+^CCR9^+^ co-expressing subset shows the highest expression of activation markers, such as PD-1 and ICOS, among memory and effector CD4^+^ T cells, indicating they are more likely to interact with B cells [11]. Collectively, these findings highlight a multi-subset T cell network contributing to B cell activation in SjD, with CCL25–CCR9 interactions representing a central pathway for glandular immune recruitment [7].

Evidence from experimental models supports the role of the CCL25–CCR9^+^ Th axis in glandular immunopathology [7]. In NOD mice, CCR9^+^ IL-21^+^ CD4^+^ T cells are enriched in the pancreas and salivary glands, and migration into inflamed tissues is shown to be CCL25-dependent [7]. Treatment with an anti-CCL25 monoclonal antibody reduced CCR9^+^ T cell migration into the pancreas and salivary glands, decreased tissue infiltration and insulitis, and prevented the onset of autoimmune diabetes [7]. Similarly, in a skin graft model, CCL25 blockade prolonged graft survival and suppressed CCR9^+^ T cell-mediated inflammation [12]. The NOD.H2(h4) mouse model spontaneously develops SjD-like disease, including salivary gland infiltration, anti-Ro/La autoantibody production, and the formation of ectopic germinal centers between 12 and 16 weeks of age [13,14]. This mouse model provides a platform to test therapeutic interventions that target lymphoid neogenesis [13,14]. In this study, we treated NOD.H2(h4) mice with an anti-CCL25 monoclonal antibody to evaluate whether blocking CCR9^+^ T cell recruitment to the salivary glands could reduce EGC formation and local autoantibody production in this model of SjD.

## 2. Results

### 2.1. Treatment Tolerance

To assess the tolerability of α-CCL25 treatment, we monitored mouse body weight throughout the 12-week study period. All treatment groups—including α-CCL25, isotype control, and PBS, demonstrated steady weight gain over time, with no significant differences in weight observed between groups (Supplementary Figure S1). At baseline (week 0), the mean body weights were comparable across groups: α-CCL25 (21.0 g), isotype control (20.9 g), and PBS (21.3 g). By week 12, mice in all three groups had gained weight, with final averages of 24.1 g (α-CCL25), 24.0 g (isotype), and 24.1 g (PBS). All animals maintained healthy weights throughout, individual weight data for all mice at baseline and at weeks 4, 8, and 12 are provided in Supplementary Table S1. These findings indicate that chronic administration of α-CCL25 was well tolerated and did not result in systemic toxicity or growth suppression.

### 2.2. Saliva Production and Gland Function

**Figure 1:**
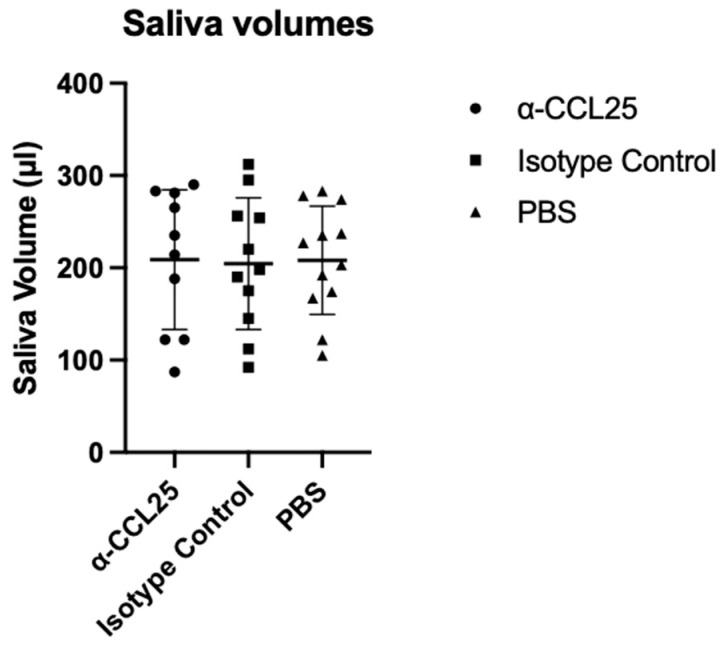
Salivary output in NOD.H2(h4) mice following 12 weeks of treatment. Saliva volumes were measured at the study endpoint following pilocarpine stimulation in NOD.H2(h4) mice treated with anti-CCL25 monoclonal antibody, isotype control IgG2b, or vehicle (PBS). Saliva was collected over a 15 min period and volume was determined gravimetrically by comparing pre- and post-collection swab weights. Each dot represents an individual mouse; horizontal lines indicate group medians. Statistical analysis was performed using one-way ANOVA, which showed no significant difference in saliva volume across treatment groups (F (2,30) = 0.012, *p* = 0.9879).

Stimulated saliva production was used as a readout of glandular function across treatment groups. Despite variability among individual mice, median saliva volumes were comparable in the α-CCL25, isotype control, and PBS groups. One-way ANOVA confirmed no significant differences in salivary output between groups (F (2,30) = 0.012, *p* = 0.9879; Figure 1). Notably, one mouse in the α-CCL25 cohort produced no detectable saliva, and a few high responders were observed in each group. Supplementary Table S2 provides individual saliva volumes, along with corresponding pilocarpine and anesthetic doses. These data suggest that CCL25 blockade does not significantly impact stimulated salivary gland function in NOD.H2(h4) mice.

### 2.3. Histopathological Assessment of Salivary Glands

**Figure 2:**
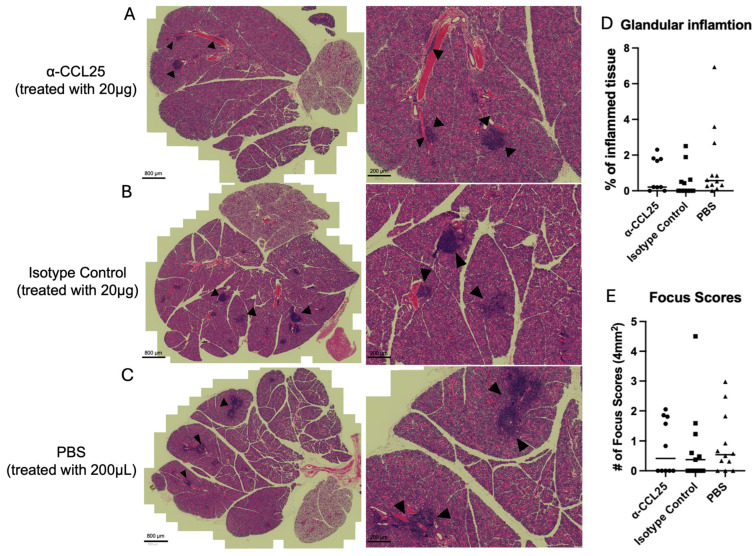
α-CCL25 treatment does not significantly alter lymphocytic infiltration in salivary glands. Representative H&E-stained submandibular salivary gland sections from mice treated with (**A**) α-CCL25 (20 μg), (**B**) isotype control (20 μg), or (**C**) PBS (200 μL). Black arrowheads indicate lymphocytic foci. Scale bars = 800 μm (left panels) and 200 μm (right panels). Right: Quantification of salivary gland inflammation using two measures (**D**) percentage of total tissue area occupied by inflammatory infiltrates, and (**E**) focus scores, defined as the number of lymphocytic foci per 4 mm^2^ of glandular tissue. Kruskal–Wallis analysis of inflammation percentage showed no significant differences across groups (statistic = 3.121, *p* = 0.2100). One-way ANOVA revealed also no significant differences in focus scores among treatment groups (F = 0.020, *p* = 0.980). Each point represents an individual mouse; horizontal bars indicate group medians.

Lymphocytic infiltration in the salivary glands remained highly variable and was not significantly impacted by α-CCL25 treatment. The percentage of glandular tissue occupied by inflammatory aggregates showed overlapping distributions across all treatment groups, with no consistent reduction in the α-CCL25 cohort (Figure 2, Supplementary Table S3). Kruskal–Wallis analysis confirmed no significant differences in inflammation percentages among groups (statistic = 3.121, *p* = 0.2100). Focus scores also did not differ significantly between treatment groups (F = 0.020, *p* = 0.980; Figure 2). Representative H&E-stained sections illustrate a wide range of infiltration distributions within each group, including both minimal and substantial lymphocytic involvement (Figure 2). These results suggest that CCL25 blockade did not significantly alter histopathological features of inflammation in the salivary glands. The lack of effect may reflect redundancy in chemokine-mediated recruitment pathways or limited tissue penetration of the antibody via systemic administration.

### 2.4. Detection of Anti-Ro52 Autoantibodies in Plasma and Saliva

Anti-Ro52 autoantibodies were undetectable in both plasma and saliva of NOD.H2(h4) mice across all treatment groups and timepoints. Indirect ELISAs were performed on plasma collected at baseline, mid-treatment, and study endpoint, as well as on saliva collected at the endpoint. In all cases, optical density (OD) values were indistinguishable from those of negative controls and remained well below the threshold for positivity. No temporal trends or treatment-related differences were observed. Although the NOD.H2(h4) strain has previously been reported to develop Sjögren’s-like features [13], the absence of detectable anti-Ro52 antibodies in this cohort suggests that the serologic profile associated with classical SjD may not be fully recapitulated under the conditions of this study. This finding limits the utility of anti-Ro52 as a readout for treatment efficacy in this model.

## 3. Discussion

In this study, we investigated the therapeutic potential of α-CCL25 antibody treatment in a spontaneous model of SjD using female NOD.H2(h4) mice. Despite successful long-term administration and good tolerability, α-CCL25 treatment did not significantly alter salivary gland inflammation, focus scores, or stimulated saliva production compared to isotype and vehicle controls. Additionally, anti-Ro52 autoantibodies were undetectable in both plasma and saliva at all timepoints, regardless of treatment group. These findings suggest that systemic blockade of CCL25 alone may be insufficient to resolve or prevent glandular inflammation in this model.

The absence of significant treatment effects on salivary gland inflammation or function suggests that CCL25–CCR9 signaling, while implicated in T cell recruitment, may not be the sole driver of immune infiltration in this spontaneous Sjögren’s model. Several possibilities could account for the observed lack of therapeutic response. First, redundancy among chemokine pathways may allow compensatory recruitment of lymphocytes via alternative signals, such as CXCL13 or CCL19, which are known to contribute to lymphoid organization in the salivary glands [15]. Second, although the antibody was delivered systemically at a biologically relevant dose, local concentrations within the inflamed salivary tissue may have been insufficient to fully block CCR9^+^ cell trafficking. Third, it is possible that the timing of treatment, initiated prior to disease onset, was too early or poorly matched to the kinetics of glandular infiltration, thereby limiting the opportunity to observe a therapeutic effect. Additionally, the variability in histological outcomes across all groups, including several mice with minimal or no detectable inflammation, may have further reduced the statistical power to detect modest treatment effects. Together, these factors highlight the complexity of modulating immune cell trafficking in established autoimmune environments and suggest that monotherapy targeting CCL25 may not be sufficient in isolation.

Despite screening plasma and saliva at multiple timepoints, anti-Ro52 autoantibodies were not detected in any of the NOD.H2(h4) mice in this study. This result stands in partial contrast to previous reports suggesting that this strain develops a Sjögren’s-like phenotype, including immune infiltration and occasional seropositivity [13,14,16]. While we did observe lymphocytic infiltration consistent with focus scores in many mice, the overall extent of inflammation and functional impact (e.g., salivary output or autoantibody levels) appeared milder than anticipated in some animals. The absence of anti-Ro52 in both biofluids suggests that, under the conditions used here, the serological features of SjD were not recapitulated. This may reflect variability in disease expression among NOD.H2(h4) colonies, differences in housing or microbiome influences [17,18], or the possibility that disease in this model is primarily gland-limited and does not reliably progress to systemic autoantibody production [19]. Because anti-Ro52 is a hallmark biomarker of human SjD [20], its absence in this cohort limits our ability to assess the effects of α-CCL25 monoclonal antibody treatment on systemic autoimmunity. On the other hand, we have used different techniques from previous studies to detect anti-Ro [14,21]. Thus, this difference could be related to varying assays and techniques. Nonetheless, these findings also highlight a broader challenge in preclinical SjD research, which is the need for better-characterized models that consistently reflect both local and systemic aspects of the disease. While the NOD.H2(h4) model remains valuable for studying glandular inflammation, it may not be optimal for tracking Ro52-specific B cell responses or testing interventions aimed at modulating autoantibody production.

Previous studies have shown evidence that CCR9^+^ T cells are enriched in salivary gland infiltrates from SjD patients and may contribute to EGC formation [7]. Other studies reported elevated CCL25 expression in the inflamed salivary glands of SjD patients, along with infiltrations of CCR9^+^CD4^+^ T cells [8]. However, in the present study, systemic blockade of CCL25 failed to reduce salivary gland inflammation or alter lymphoid architecture, suggesting that this chemokine alone may not be sufficient to drive or sustain chronic immune infiltration. The discrepancy may reflect tissue-specific differences in chemokine hierarchy, the stage of disease at intervention, or the unique features of the NOD.H2(h4) model. Our data align more closely with recent evidence that targeting a single chemokine axis may be inadequate to reverse established tertiary lymphoid structures or functional gland damage, particularly when multiple redundant pathways are active [22]. Together, this comparison underscores the complexity of chemokine networks in chronic autoimmunity and the need to consider combinatory approaches or model-specific features when evaluating immunomodulatory strategies.

Several factors may have limited the therapeutic impact of α-CCL25 treatment in this study. First, although the antibody was administered systemically at a biologically relevant dose, it is unclear whether sufficient levels reached the salivary gland microenvironment to effectively block CCR9-mediated recruitment. Local delivery methods or tissue-level pharmacokinetic analysis may be necessary in future studies to confirm effective target engagement. Direct and repeated injections into the salivary glands can induce local inflammation and fibrosis, which may interfere with downstream immunohistochemistry and histopathological evaluation of the tissue. To address this, we performed ELISAs on both saliva and plasma, but CCL25 was not detectable in either fluid. More detailed biodistribution analyses (e.g., Western blotting or immunohistochemistry) were not feasible within the scope of this study, and we have noted this as a limitation and as an important avenue for future work. Second, treatment was initiated at a relatively early stage, prior to the appearance of overt glandular infiltration. It is possible that intervening at a different disease stage, either earlier to prevent infiltration or later to disrupt established inflammation, may yield different outcomes. Additionally, the NOD.H2(h4) model showed considerable variability in disease expression within and across treatment groups. Several mice had little to no detectable inflammation or focus formation, while others showed more advanced pathology. This heterogeneity may have reduced statistical power to detect modest treatment effects. Furthermore, the absence of detectable anti-Ro52 autoantibodies limits the model’s utility for evaluating systemic serologic autoimmunity and suggests that immune activation in this setting may be largely gland restricted. In addition, we did not directly assess ectopic germinal centers (EGCs). As all three groups exhibited comparable levels of inflammation, further immunohistochemistry was unlikely to provide discriminatory insight; however, the absence of EGC evaluation remains a limitation of the present work. The possibility of chemokine pathway redundancy remains an important consideration, as targeting a single axis such as CCL25 may not be sufficient to interrupt chronic inflammation driven by overlapping recruitment signals. Finaly, future studies may benefit from exploring the use of bivalent antibodies, which could enhance efficacy and provide a stronger blockade of the CCL25–CCR9 axis.

This study evaluated the therapeutic potential of α-CCL25 antibody treatment in a spontaneous mouse model of Sjögren’s disease. These findings suggest that CCL25 blockade alone is insufficient to reverse or prevent glandular pathology in the NOD.H2(h4) strain, potentially due to limited tissue penetration, chemokine pathway redundancy, or disease heterogeneity. Although the CCL25–CCR9 axis remains biologically relevant, future strategies may require combination therapies, improved delivery methods, or alternative models to fully assess its therapeutic utility in SjD.

## 4. Materials and Methods

### 4.1. Animal Model

We obtained 34 female NOD.H2(h4) mice (6–8 weeks old; stock number 004447) from Jackson Laboratories (Bar Harbor, ME, USA). Experiments were conducted on female mice only to reflect the predominance of SjD in women (by more than 9:1) [1]. In addition, female NOD.H2(h4) mice are known to exhibit more severe salivary gland infiltration, elevated autoantibody production, and enhanced B cell activation compared to males, making them the preferred sex for modeling SjD-like disease manifestations [21]. Mice were housed in a specific pathogen-free facility in the Oklahoma Medical Research Foundation vivarium (3–5 animals/cage). All mice had unrestricted access to food (irradiated pelleted 5053 from LabDiet, St. Louis, MO, USA) and water. All animal experiments were approved by the Institutional Animal Care and Use Committee and followed the National Institutes of Health’s guidelines for the care and use of laboratory animals.

### 4.2. Blood Collection and Processing

**Figure 3:**
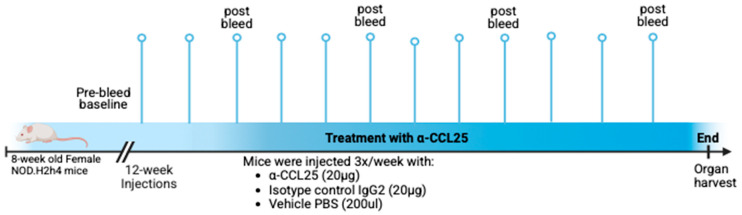
Overview of the 12-week experimental design in female NOD.H2(h4) mice beginning at 8 weeks of age. This schematic illustrates the experimental timeline and treatment groups. Mice received a baseline pre-bleed prior to treatment initiation, followed by intraperitoneal injections three times per week for 12 weeks with either anti-CCL25 monoclonal antibody (20 µg), isotype control IgG2b (20 µg), or vehicle (200 µL PBS). Peripheral blood was collected biweekly starting at week 12, and mice were monitored throughout the study. At the end of the treatment period, saliva was collected and organs were harvested for downstream analysis. Created in BioRender (https://BioRender.com).

Peripheral blood was collected from all mice via tail vein sampling, beginning with a baseline pre-bleed at 8 weeks of age, prior to starting treatment. Subsequent collections were performed every 3 weeks, starting at 12 weeks of age, and continued throughout the 12-week treatment period. At each timepoint, mice were weighed prior to blood collection to monitor health status and to allow normalization of select analytes to body weight when applicable (Figure 3, Supplementary Table S1 and Figure S1). Each mouse tail was briefly warmed under a heat lamp to facilitate vasodilation of the tail veins and then placed in acrylic mouse restrainers. The lateral tail vein was punctured using a sterile 25-gauge needle, and approximately 70–100 µL of blood collected in heparinized 70 µL capillary tubes was transferred into 1.5 mL microcentrifuge tubes. After sampling, hemostasis was achieved by applying gentle pressure with a swab. Blood was processed by centrifugation at 12,000× *g* for 10 min and plasma was stored at −80 °C for later analysis (Figure 3).

### 4.3. Treatments—Dosing and Administration

Mice were treated via intraperitoneal (IP) injections using sterile 25-gauge needles three times per week for a total duration of 12 weeks. The animals were divided into three treatment groups (Figure 3). Eleven mice received 20 µg of anti-CCL25 monoclonal antibody per dose (mouse CCL25/TECK antibody, monoclonal Rat IgG2_B_; clone #89818; Bio-Techne, R&D Systems, Minneapolis, MN, USA), eleven mice received 20 µg of isotype control antibody per dose (monoclonal Rat IgG2b isotype control; clone #141945; Bio-Techne, R&D Systems), and twelve mice received vehicle control (1× PBS; pH 7.4; Thermo Scientific (Waltham, MA, USA), cat. #28372). Antibody solutions were freshly prepared on the day of each injection. The anti-CCL25 antibody was prepared by diluting 82 µL of stock solution (9.24 mg/mL) in sterile 1× PBS, pH 7.4. The isotype control was prepared by diluting 68.5 µL of stock solution (11.06 mg/mL) in 1× PBS, pH 7.4. All antibody stock solutions were stored at −80 °C and, once thawed, were kept at 4 °C and used until depleted. The injection volume was 200 µL for each mouse (0.1 μg/μL).

### 4.4. Saliva Collection

Saliva was collected from all mice on the final day of the study, prior to euthanasia and organ harvesting. The procedure was adapted from a previously described protocol [23]. Mice were anesthetized with an intraperitoneal injection of an anesthetic cocktail (ketamine (100 mg/kg) and xylazine (8 mg/kg) in saline 0.9%NaCL; dose based on body weight, Supplementary Table S2), and ophthalmic ointment was applied to prevent corneal drying. Pilocarpine hydrochloride was administered intraperitoneally at a dose of 0.375 mg/kg to stimulate salivary secretion. Each mouse was placed in a restraining tube at a 45° angle with the ventral side up, and a pre-weighed conical swab (SalivaBio Children’s Swab, Salimetrics, State College, PA, USA) was gently inserted into the oral cavity for 15 min. Following the collection period, the swab was removed, placed into a pre-weighed 0.6 mL microfuge tube nested within a 2 mL tube, and stored on ice. The saliva weight was determined by calculating the difference between the wet and dry swab weights (Supplementary Table S3). Samples were then centrifuged at 7500× *g* for 2 min at 4 °C, and saliva volume was recovered using a micropipette and measured. Saliva output was normalized either by time and body weight and recorded as mg/15 min or mg/g body weight, respectively. Immediately following saliva collection, mice were euthanized using CO_2_ as the primary method and cervical dislocation as the secondary (confirmatory) method. Tissues were then harvested for downstream analyses. One mouse in the α-CCL25 cohort died during the last week of this study for unknown reasons; tissues and blood were collected but no saliva.

### 4.5. Organ Harvesting and Tissue Processing

At the study endpoint, the mice were euthanized, and salivary glands (submandibular and parotid) were carefully dissected and connective tissue removed. Formalin-fixed, paraffin-embedded (FFPE) salivary glands were sectioned (5 μm) and stained with hematoxylin and eosin (H&E) prior to evaluation for inflammatory infiltrates. High-resolution whole-slide images were acquired using a Zeiss AxioScan 7 digital pathology scanner (Minneapolis, MN, USA), and image analysis was performed using QuPath software (v0.6.0) [24]. A lymphocytic focus was defined as an aggregate of ≥50 mononuclear cells within the glandular parenchyma. Focus scores were calculated as the number of foci per 4 mm^2^ of tissue. In addition to scoring, the area of inflammation was quantified as the percentage of total parenchymal area involved, using the formula (area of lymphocytic foci/total gland area) × 100 [25].

### 4.6. ELISA—Autoantibody Screening Assays

Whole saliva and plasma samples were screened for anti-Ro52 (IgG) antibodies using direct enzyme-linked immunosorbent assays (ELISAs) developed in-house. ELISAs were performed on Enhanced Binding Immuno Breakable Module plates (ThermoFisher, Waltham, MA, USA), which were coated overnight at 4 °C with 100 μL per well of Ro52 antigen (MBP-mRo52; 1 μg/mL) in carbonate–bicarbonate coating buffer, pH 9.6. Plates were then washed twice with phosphate-buffered saline and Tween-20 (0.05%) (PBS-T) and blocked with 1% bovine serum albumin (BSA, IgG-free) in PBS for one hour at room temperature. Saliva (diluted 1:25) and plasma (diluted 1:100) samples were run in duplicate and incubated for two hours at room temperature. After 5 rounds of washing, bound antibodies were detected using a peroxidase-conjugated, affinity-purified goat anti-mouse IgG secondary antibody (#115-035-166; Jackson ImmunoResearch, West Grove, PA, USA), incubated for one hour at room temperature. o-Phenylenediamine dihydrochloride (OPD) was used as the chromogenic substrate, and the reaction was stopped with 2.5 N sulfuric acid solution after 15 min incubation. Plates were read at 490 nm, and to correct for nonspecific absorbance, the optical density (O.D.) at 650 nm was subtracted from the substrate-specific O.D. at 490 nm. Final O.D. values were adjusted by subtracting the blank adjusted O.D. For normalization, each sample’s mean O.D. was divided by the mean O.D. of the positive control on the same plate. A sample was considered positive if its normalized O.D. value was higher than the pre-bleed mean normalized O.D. plus three standard deviations (mean + 3 SD).

### 4.7. Statistical Analysis

All statistical analyses were performed using GraphPad Prism software (version 9.4, GraphPad Software, San Diego, CA, USA) and Microsoft Excel (version 19.89.1, Microsoft Corporation, Redmond, WA, USA). Descriptive statistics were used to summarize body weight, saliva production, and histopathology scores. Assuming approximate normality, one-way ANOVA was used to compare salivary flow and focus scores across the three treatment groups (α-CCL25, isotype control, and PBS). The distribution of inflammation percentages was skewed and non-normal, so the Kruskal–Wallis test was used to compare the inflammation area among groups. When applicable, post hoc analyses were performed to explore pairwise differences. Statistical significance was assessed using two-tailed *p*-values, with α set at 0.05. *p*-values < 0.05 were considered statistically significant.

## Data Availability

Data is contained within the article and Supplementary Materials.

## Supplementary Materials

The following supporting information can be downloaded at: https://www.mdpi.com/article/10.3390/ijms26188802/s1.
