# Supplementary material for: Evaluating Anti-CCL25 as a Therapeutic Strategy to Disrupt Foci Formation in a Spontaneous Murine Model of Sjögren’s Disease
Source: Int J Mol Sci. 2025 Sep 10;26(18):8802. doi: 10.3390/ijms26188802 (PMC12470085; doi:10.3390/ijms26188802)
Supplement: Supplementary file 1 [file ijms-26-08802-s001.zip › ijms-3838072-supplementary.pdf]

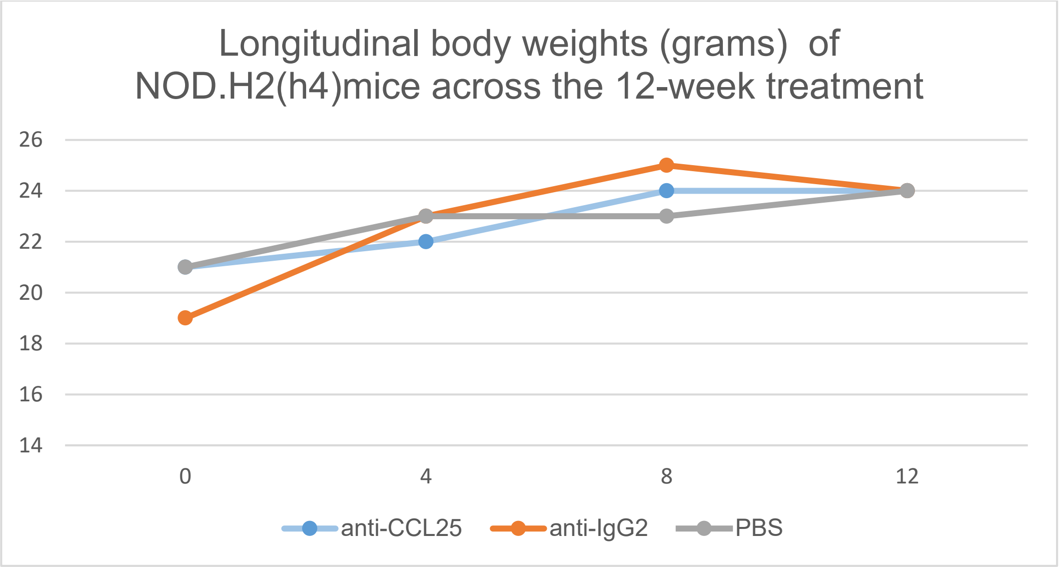

Supplementary Figure S1: Longitudinal body weights of NOD.H2(h4) mice by treatment group over 12 weeks. Average body weights (in grams) of mice treated with anti-CCL25 monoclonal antibody, isotype control (IgG2b), or vehicle control (PBS) were recorded at baseline (week 0), and at weeks 4, 8, and 12 during the 12-week treatment period. Each line represents the mean weight for mice in the respective treatment group at each timepoint. Although minor fluctuations were observed, all groups exhibited a general trend of weight gain over the course of the study, indicating maintained overall health throughout treatment.

Supplementary Table S1: Longitudinal body weights of NOD.H2(h4)mice across the 12-week treatment period. Body weights (in grams) of individual NOD.H2(h4)mice recorded throughout the study. Mice were weighed at four timepoints: baseline (prior to treatment initiation), and then every four weeks during the 12-week treatment period. "1st weight" corresponds to pre-treatment baseline (week 0), followed by "2nd," "3rd," and "4th" weights taken at approximately weeks 4, 8, and 12, respectively. Data were used to monitor overall health status and to normalize pilocarpine treatment and saliva production. Died*: One mouse in the anti-CCL25 treatment cohort (Mouse #7) died prior to the final weight collection.

Mice	1st weight (grams)	2nd weight (grams)	3rd weight (grams)	4th weight (grams)
1	21.9	22.9	24.7	25.4
2	16.5	19.6	20.6	20.5
3	20.2	22.8	24.4	23
4	19.6	21.8	21.4	21.7
5	22.6	24.8	25.5	24.7
6	20.9	22.1	24.8	24.5
7	18.2	22.9	26.1	Died*
8	20.6	24.4	25.5	27.4
9	23.9	24.9	26.8	26.2
10	19.3	22.5	25.1	24.1
11	20.8	22.2	24.6	24.6
12	18.2	22	23.5	21.8
13	20	23.2	23.5	24.7
14	18.5	21.7	22.8	22.9
15	19.2	21.4	22	23.1

16	20.6	22.5	24.1	25.1
17	20.4	21.4	22.8	22.8
18	19.2	20.6	22	22
19	20.3	22.6	24	25
20	21.8	23.6	23.9	25.1
21	21.3	24.4	24.5	25
22	22	26.1	25.8	27.1
23	20	22.5	22.5	23.9
24	20.4	22.1	24.3	22.8
25	22	25.3	24.3	25.6
26	23.5	25.5	24.1	23.6
27	20.5	22.6	24.2	23.6
28	20.3	26.5	22.8	23.6
29	20.8	23.8	26.5	27
30	10	21.3	26.5	22.7
31	20.6	21.2	23.3	24.2
32	22.6	21.5	22.6	24
33	21.9	24.7	24.2	24.1
34	19.1	21.3	21.8	22.7

Supplemental Table S2: Saliva collection parameters and volumes for individual NOD.H2(h4)mice at endpoint. Saliva was collected from each mouse on the final day of the study following anesthesia and stimulation with pilocarpine. The ketamine cocktail volume (µL) was calculated based on individual body weight, as was the volume of pilocarpine hydrochloride administered (0.375 mg/kg). Saliva was collected for 15 minutes post-injection and measured in microliters (µL) after centrifugation. One mouse (Mouse #33) did not produce measurable saliva. Data were used to assess functional salivary output in relation to treatment response. Died*: One mouse in the anti-CCL25 treatment cohort (Mouse #7) died prior to the final weight collection.

Mouse	Weight (grams)	Ketamine cocktail (µl)	Pilocarpine (µl)	Saliva volume (µl)
1	25.4	228.6	170	290
2	20.5	206	138	87
3	23	230	154	188
4	21.7	195	145	122
5	24.7	222	165	281
6	24.5	221	161	235
7	Died*	Died*	Died*	Died*
8	27.4	274	184	92
9	26.2	235.8	175	220
10	24.1	241	162	145
11	24.6	221.4	165	112
12	21.8	196	146	198
13	24.7	222	165	256
14	22.9	206	153	175
15	23.1	208	155	237
16	25.1	226	168	235

17	22.8	205	152	278
18	22	198	147	203
19	25	225	167	192
20	25.1	226	168	227
21	25	250	168	105
22	27.1	244	181	174
23	23.9	215.1	160	122
24	22.8	205	153	214
25	25.6	230	171	265
26	23.6	212	158	283
27	23.6	212	158	254
28	23.6	212	158	312
29	27	243	181	295
30	22.7	204	152	190
31	24.2	218	162	167
32	24	216	161	274
33	24.1	217	161	No Saliva
34	22.7	204	152	283

Supplemental Table S3: Quantification of salivary gland inflammation in NOD.H2(h4)mice following 12 weeks of treatment with anti-CCL25 monoclonal antibody, isotype control (IgG2b), or vehicle control (PBS). At endpoint, formalin-fixed, paraffin-embedded salivary gland sections (5 μm) were stained with hematoxylin and eosin (H&E). High-resolution digital slides were analyzed using QuPath software to measure total glandular area (Sum of SG tissue, in μm²) and the area occupied by dense lymphocytic aggregates (Sum of lymphocyte, in μm²). The percentage of inflammation was calculated as: (area of lymphocyte infiltration / total gland area) × 100. Samples in which no discrete lymphocytic foci were detected are marked as "No inflammation detected.

α-CCL25 treated			
**# Mouse**	**Sum of SG tissue (μm²)**	**Sum of lymphocyte (μm²)**	**% of inflammation**
Mouse 1	11186960	23648.3	0.211391656
Mouse 3	9783625.56	164053.4	1.676816013
Mouse 4	10114084.7	21005.6	0.207686614
Mouse 5	12448300.37	222123.6	1.784368897
Mouse 6	8999204.583	17433.7	0.193724899
Mouse 7	7774184	No inflammation detected	No inflammation detected
Mouse 23	14840789	341650.4	2.302104019
Mouse 24	11236022.5	No inflammation detected	No inflammation detected
Mouse 25	14893484.6	270474.5	1.816059218
Mouse 26	13386756.4	No inflammation detected	No inflammation detected
**Isotype Control**			
**# Mouse**	**Sum of SG tissue (μm²)**	**Sum of lymphocyte (μm²)**	**% of inflammation**
Mouse 8	10750685	67215.5	0.625220625

Mouse 9	12437975	311537.3	2.504726855
Mouse 10	6725538.5	33855.4	0.503385714
Mouse 11	8716489.5	No inflammation detected	No inflammation detected
Mouse 12	10562069.5	No inflammation detected	No inflammation detected
Mouse 13	109761129.1	No inflammation detected	No inflammation detected
Mouse 14	9456369.1	No inflammation detected	No inflammation detected
Mouse 27	158953886.6	67161.3	0.431622874
Mouse 28	16658392.4	315110.8	1.891603898
Mouse 39	10398433.8	No inflammation detected	No inflammation detected
Mouse 30	344022534.7	No inflammation detected	No inflammation detected
**PBS vehicle**			
**# Mouse**	**Sum of SG tissue (μm²)**	**Sum of lymphocyte (μm²)**	**% of inflammation**
Mouse 15	12444538.87	333671.8	2.681270905
Mouse 16	10822050.68	36517.1	0.337432351
Mouse 17	7899362.9	67940.6	0.860076956
Mouse 18	7355121.3	No inflammation detected	No inflammation detected
Mouse 19	12211303.7	39549.3	0.32387451
Mouse 20	11986552	430189.2	3.588931996
Mouse 21	12291282.4	13251.9	0.107815438
Mouse 22	8867587.3	54207.7	0.611301566
Mouse 31	1118927.942	537198.1	0.851851102
Mouse 32	1118927.942	77636.9	6.938507576
Mouse 33	11345700.2	31011.1	0.273329098
Mouse 34	5122653.9	27128.2	0.530
